# Clinical, Functional, and Psychosocial Profiles of Chronic Obstructive Pulmonary Disease (COPD) Etiotypes: A Taxonomy-Based Analysis

**DOI:** 10.3390/medicina62020348

**Published:** 2026-02-09

**Authors:** Irem Altan, Fatih Alasan, Ozlem Sengoren Dikis, Fulden Cantas Turkis

**Affiliations:** 1 Faculty of Medicine, Department of Chest Diseases, Mugla Sıtkı Koçman University, 48000 Mugla, Türkiye; iremaltan@mu.edu.tr (I.A.); ozlemsengoren@mu.edu.tr (O.S.D.); 2 Faculty of Medicine, Department of Medical Biostatistics, Mugla Sıtkı Koçman University, 48000 Mugla, Türkiye; fuldencantas@mu.edu.tr

**Keywords:** chronic obstructive pulmonary disease, GOLD 2023 taxonomy, etiotypes, phenotyping, pulmonary function, radiology, psychosocial status, precision medicine

## Abstract

*Background and Objectives*: The 2023 GOLD report introduced seven etiological categories, known as etiotypes, to better reflect the heterogeneity of chronic obstructive pulmonary disease (COPD). However, the clinical, functional, radiological, and psychosocial characteristics associated with these etiologies remain insufficiently defined. This study aimed to explore the differences among GOLD 2023 etiotypes in a stable COPD cohort. *Materials and Methods*: This prospective, observational, cross-sectional study included 315 stable outpatients with COPD from a tertiary clinic between June and July 2025. Etiological classification was based on predefined criteria, including genetic predisposition, impaired lung development, exposure-related mechanisms, infection-related mechanisms, and asthma-like characteristics. Patients were evaluated using clinical instruments (mMRC and CAT), psychological assessments (LCQ, BDI, BAI, and CAFS), pulmonary function tests, and thoracic CT scans. *Results*: COPD due to environmental exposure (COPD-E) was the most common type (98.7%), followed by infection-related (COPD-I, 13.3%), asthma-related (COPD-A, 9.8%), and combined forms (COPD-D and COPD-G, each 2.5%). Participants with COPD-A were younger (median 54 vs. 66 years; *p* < 0.001), reported less tobacco exposure (36 vs. 50 pack-years; *p* < 0.001), and showed less CT-detected emphysema (31.6% vs. 78.3%; *p* < 0.001). COPD-G exhibited more severe airflow obstruction (FEV_1_ 25.5% predicted; *p* < 0.001), higher symptom burden (CAT score 21 vs. 6; *p* < 0.001), and lower oxygen saturation (*p* = 0.001). Eosinophil counts and psychosocial measures did not significantly differ by etiology. *Conclusions*: The GOLD 2023 COPD etiotypes demonstrated distinct clinical and functional profiles, reflecting diverse underlying mechanisms of the disease. Recognizing these etiological differences can help clinicians tailor diagnostic evaluations, guide individualized treatment strategies, and ultimately improve patient outcomes. Understanding disease etiology remains a cornerstone for accurate diagnosis, personalized management and optimized therapeutic decisions in COPD.

## 1. Introduction

Chronic obstructive pulmonary disease (COPD) is a leading cause of morbidity and mortality worldwide. It is characterized by persistent respiratory symptoms and airflow limitation due to airway and/or alveolar abnormalities and is usually caused by significant exposure to noxious particles or gases [1]. Although cigarette smoking has traditionally been regarded as the primary cause of COPD, accumulating evidence indicates that the disease arises from multiple biological and environmental factors. These include impaired lung development, genetic predisposition, exposure to biomass fuels and air pollutants, infection-mediated lung injury, and asthma-associated inflammatory pathways [2]. This expanded etiological spectrum challenges the traditional concept of COPD as a single disease entity and highlights the need for classification systems that better reflect the underlying pathogenic mechanisms [1,2,3,4].

In clinical practice, COPD is primarily diagnosed by the presence of persistent airflow limitation due to a post-bronchodilator FEV_1_/FVC ratio < 0.70, owing to its simplicity and reproducibility. Disease severity has historically been categorized using GOLD spirometric grades (I–IV) and, more recently, complemented by symptom and exacerbation-based ABE classifications to guide therapeutic decisions [5]. While these approaches are useful for prognostication and treatment planning, they largely describe downstream functional impairment rather than the etiological drivers of disease development and progression [5,6].

To address these limitations, the GOLD 2023 report introduced a novel etiological taxonomy for COPD based on seven different etiotypes: genetically determined (COPD-G), developmental-related (COPD-D), smoking-related (COPD-C), biomass/pollution-related (COPD-P), infection-related (COPD-I), asthma-related (COPD-A), and unclassified (COPD-U). In clinical settings, smoking and biomass/pollution exposure often coexist in patients; therefore, these categories are commonly grouped as COPD related to environmental exposure (COPD-E) [7]. This framework conceptualizes COPD as a spectrum of pathophysiologically distinct but often overlapping endotypes, potentially associated with different clinical presentations, radiological patterns, inflammatory profiles, and therapeutic implications [8,9].

Concurrently, advances in precision medicine have further emphasized the importance of etiological classification of COPD. Accumulating data suggest that COPD is characterized by marked biological heterogeneity, shaped by complex gene–environment interactions, diverse molecular biomarkers, and multiple inflammatory pathways [4]. In this context, pathogenic pathways denote the biological mechanisms by which various etiological exposures result in lung injury and the progression of disease. These pathways may encompass exposure-related oxidative and inflammatory injury, infection-associated airway damage, asthma-related type 2 inflammation, impaired lung development, and genetic susceptibility. Notably, these mechanisms are not mutually exclusive and often coexist within individual patients, thereby contributing to the heterogeneity of clinical presentation and disease burden [5,9,10,11].

Similarly, the treatable traits paradigm advocates for the integration of etiological and phenotypic information to enable individualized treatment strategies, underscoring the need for multidimensional disease assessment beyond conventional spirometric categorization [4,10,11]. Consistent with this approach, the GOLD framework links distinct pathogenic pathways to early disease recognition, personalized therapeutic strategies, and precision-based disease management, whereby diagnostic and treatment priorities are tailored according to the dominant etiological drivers rather than airflow limitation alone [2,9,12].

Despite these conceptual advances, robust clinical evidence supporting the practical utility of etiological stratification in COPD is limited. Although previous studies have suggested that disease endotypes may differ in terms of radiological features, systemic inflammation, exacerbation risk, and treatment response, comprehensive real-life evaluations incorporating symptom burden, lung function, imaging findings, and psychosocial dimensions are lacking. Moreover, the clinical relevance of overlapping etiological characteristics commonly encountered in routine outpatient populations has not been sufficiently explored [2,9,11,13].

The objective of the present study was to address these gaps by comprehensively characterizing symptom burden, pulmonary function, radiological findings, and psychosocial status in patients with stable COPD, classified according to the GOLD 2023 cause-based taxonomy. Symptom burden was assessed using the Modified Medical Research Council (mMRC) dyspnea scale and the COPD Assessment Test (CAT), while psychosocial status was evaluated using the Leicester Cough Questionnaire (LCQ), Beck Depression Inventory (BDI), Beck Anxiety Inventory (BAI), and COPD-Asthma Fatigue Scale (CAF-S). By identifying etiotype-specific patterns and their overlap, this study aims to contribute to the growing evidence supporting etiotype-informed and personalized management approaches in COPD [14,15,16,17,18,19].

In particular, the following three research questions were considered:

1-Do 2023 GOLD etiotypes have different clinical, functional, radiological, and psychosocial characteristics?

2-Are symptoms and psychosocial outcomes significantly different between etiotypes?

3-What are the prevalence and clinical implications of overlapping etiotypes in a COPD cohort representative of real-life practices?

We aimed to test the hypothesis that etiotypes, as defined by the GOLD 2023 etiological disease assessment, would show different multidimensional clinical, functional, radiological, and psychosocial profiles. This supports the clinical relevance of etiological stratification for tailored management of COPD.

## 2. Materials and Methods

The study protocol was approved by the Ethics Committee of the Mugla Sitki Kocman University Faculty of Medicine (21 May 2025; decision no. 98). All study procedures were conducted in accordance with the principles of the Declaration of Helsinki, and written informed consent was obtained from all participants prior to enrollment.

This single-center, prospective, cross-sectional study enrolled patients with stable COPD who presented to the Chest Diseases Outpatient Clinic of Mugla Sitki Kocman University Training and Research Hospital between 1 June and 31 July 2025.

**Figure 1:**
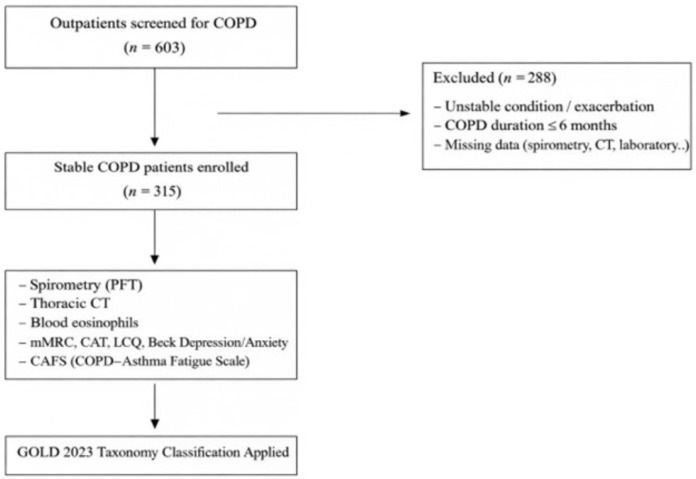
Flow diagram of the screening process and final cohort selection.

Eligible participants were required to be ≥18 years of age, have a spirometry-confirmed diagnosis of COPD established at least six months before enrollment, and remain clinically stable for a minimum of four weeks prior to inclusion. Clinical stability was defined in accordance with the ATS/ERS and SPIROMICS criteria, requiring the absence of worsening dyspnea, sputum volume, or sufficient sputum purulence to necessitate treatment escalation [20,21]. The patient screening, exclusion, and inclusion processes are shown in Figure 1.

According to the GOLD 2023 strategy, etiologies have been allocated based on pre-specified operational criteria. The GOLD guidelines specifically state that COPD-C and COPD-P often coexist in everyday practice; therefore, when both occur, a mixed environmental exposure-related etiology of COPD (COPD-E) is recorded. This dissection was consistent with the GOLD classification and was not an investigator-derived modification. As etiotypes are etiological processes rather than mutually exclusive diseases, individual patients can be assigned more than one etiotype. All classifications were independently reevaluated by two pulmonologists until a consensus was reached in cases of disagreement.

The exclusion criteria were as follows: acute exacerbation at the time of presentation, diagnosis of asthma or bronchiectasis as the primary diagnosis, active malignancy diagnosed within the previous 5 years, moderate-to-severe cardiac/hepatic/renal disease, other chronic respiratory diseases (other than COPD), or cognitive impairment that did not permit completion of psychosocial measures.

Owing to the limited number of trained physician resources, all clinical examinations were performed in person by only one physician using standard data collection forms to avoid inter-observer biases. The retrieved variables were demographic data, exposure to smoking (current status, pack-years smoked, and passive exposure), environmental factors (biomass use and occupational inhalation risk), family history of COPD, sputum features, comorbidities, anthropometric measurements (height and weight for the calculation of body mass index [BMI]), oxygen saturation, spirometric parameters (forced vital capacity [FVC], forced expiratory volume in 1 s [FEV_1_], FEV_1_/FVC ratio, and thoracic CT findings when available.

Etiotype classification was conducted using predefined criteria derived from clinical history, radiological findings, pulmonary function tests, and psychosocial assessments. Radiological evaluations were performed by experienced clinicians using standardized criteria to reduce subjectivity. Psychosocial status was evaluated using validated and widely recognized instruments (LCQ, BDI, BAI, and CAFS), which have been shown to be reliable and reproducible. To ensure consistency, all etiotype classifications were deliberated within the study team, and any discrepancies were resolved through consensus.

Spirometric severity was defined according to the GOLD criteria on a scale of I (FEV_1_ percent-predicted post-bronchodilator ≥ 80%) to IV (<30%). Additionally, patients were categorized according to the GOLD A–B–E classification based on symptoms and exacerbations (group A, low symptom burden with a low exacerbation history; group B, high symptom burden with a low exacerbation history; and group E, regardless of symptom level).

Spirometry adhered to the ATS/ERS technical standards, including daily calibrations and rigorous quality control measures. The clinical stability criteria used in the analysis were identical to those defined previously [20,21].

The modified mMRC dyspnea scale and CAT were used to assess the symptom burden [14,15]. The psychosocial status of these groups was evaluated using the LCQ, BDI, BAI, and CAFS (the Turkish version of which is valid) [16,17,18,19]. These instruments were considered because they are commonly used in chronic respiratory diseases and have strong psychometric properties across the constructs of dyspnea, health status, cough-specific QoL, depression, anxiety, and fatigue. The well-established Turkish versions provided cultural and linguistic appropriateness for the participant pool in the present study. All questionnaires were administered in separate, standardized, and quiet rooms.

The operational definitions for etiotypes were as follows.

- COPD-G (genetically determined): Known or suspected alpha-1 antitrypsin deficiency based on serum AAT levels or a family history of AAT deficiency.
- COPD-D (development-related): Prematurity (<37 weeks), low birth weight (<2.5 kg), severe childhood respiratory illness requiring hospitalization, persistent early onset asthma, or <10 pack-years of smoking without an alternative dominant etiological factor.
- COPD-C (smoking-related): ≥10 pack-years of tobacco or chronic secondhand smoke exposure.
- COPD-P (biomass/pollution-related): prolonged biomass fuel exposure, occupational inhalation hazards, or sustained indoor/outdoor air pollution exposure.
- COPD-E (environmental exposure-related): Applied when the criteria for both COPD-C and COPD-P were fulfilled, consistent with the GOLD 2023 conceptual grouping.
- COPD-I (infection-related): Previous pulmonary infections, such as tuberculosis, recurrent childhood pneumonia, or HIV-associated lung disease.
- COPD-A (asthma-associated): physician-diagnosed asthma before the age of 40 years, eosinophil counts ≥300/µL, or significant bronchodilator reversibility (>12% and >200 mL).
- COPD-U (unclassified): No etiological mechanisms were identified.

The anonymity of all the data was guaranteed throughout the process. Missing values (<5%) were deleted. All statistical analyses were conducted using R software (version 3.2.5). The normality of continuous variables was tested using the Kolmogorov–Smirnov test. Continuous normally distributed variables are expressed as mean ± standard deviation (SD), and differences between groups were compared using an independent sample *t*-test. Continuous non-normally distributed variables are presented as medians (25th–75th percentiles) and were compared using the Mann–Whitney U test. Categorical variables were described using frequencies and percentages and compared using the chi-square test. All *p*-values were two-tailed, and a value of < 0.05 was considered statistically significant. 

## 3. Results

### 3.1. Baseline Characteristics of the Study Population

In total, 315 patients with COPD were included in this study. The cohort was predominantly male (87.6%) with a mean age of 64.2 ± 10.8 years old. The 39 female patients (12.4%) had a lower mean age than male patients (61.4 ± 9.3 vs. 64.8 ± 10.9 years). Almost half of the patients were current smokers (49.8%), 42.9% were former smokers, and 5.1% had never smoked. Passive smoke exposure among never-smokers was reported in 43.8% of the cases. A family history of COPD was present in 22.5% of the population, and the majority of patients (62.5%) had moderate-to-severe airflow limitation according to the GOLD spirometric staging. Eosinophil counts ≥300/μL were found in 22.2% of patients. Thoracic CT abnormalities were observed in 95.8% of patients, most frequently emphysema, interstitial changes, and bronchiectasis. The median values of the pulmonary function and psychosocial assessments are summarized in Table 1, which presents the baseline demographic, clinical, and functional characteristics of the study population.

### 3.2. Distribution and Overlap of GOLD 2023 Etiotypes

Following cohort characterization, patients were categorized using the GOLD 2023 etiological taxonomy. The distribution and overlap of the patterns are summarized in Table 1. COPD-E was the most common cause of the disease (*n* = 311, 98.7%). The other etiologies were COPD-I (*n* = 42), COPD-A (*n* = 31), COPD-G (*n* = 8), and COPD-D (*n* = 8). All patients were classified within at least one defined etiotype, and none were categorized as having COPD-U. Etiological overlap was frequent, especially in COPD-E with COPD-I, COPD-A, and COPD-G. Very few patients had only one specific aetiotype, indicating that COPD is a multifactorial disease in this population. The aetiotype frequencies and overlapping patterns are presented in Table 2.

### 3.3. Comparison Between COPD-A and Non–COPD-A Groups

Patients with COPD-A (*n* = 31) differed from those without (*n* = 284) in terms of demographic and clinical characteristics (Table 3). Patients in the COPD-A group were significantly younger, with a median age of 54 (47–60) years versus 66 (59–72) years in the non-COPD-A group (*p* < 0.001). Laboratory values, including hemoglobin levels, eosinophil counts, and oxygen saturation, were also similar (*p* > 0.05). There were no significant between-group differences in symptom-related outcomes (mMRC, CAT, and LCQ domains and total scores, BDIScore, and CAFS). However, the median BAI score was significantly higher in the COPD-A group [6 (3–9) vs. 4 (1–7), *p* = 0.010].

### 3.4. Comparison Between COPD-G and Non–COPD-G Groups

Patients in the COPD-G group (*n* = 8) exhibited severe clinical profiles. Hypoxemia (SpO_2_ < 90%) was more frequent in this group (62.5%; *p* < 0.001), and severe airflow limitation (GOLD IV) was observed in 87.5% of patients (*p* < 0.001). Bronchiectasis was also more prevalent in the COPD-G group than in the-non–COPD-G group (42.9% vs. 10.9%, *p* = 0.038).

The comparative analyses are presented in Table 4. Patients in the COPD-G group were younger and had a lower BMI and smoking exposure (all *p* ≤ 0.039), whereas sex distribution did not differ (*p* = 0.259). Pulmonary function parameters (FVC, FEV_1_, FEV_1_/FVC, and percent predicted values) were significantly reduced in the COPD-G group (all *p* ≤ 0.028). Resting oxygen saturation was lower in the COPD-G (*p* = 0.001), whereas oxygen saturation under supplemental oxygen was similar between the groups (*p* = 0.458).

Laboratory parameters, including hemoglobin and eosinophil counts, did not differ significantly (all *p* > 0.05). Symptom burden was higher in the COPD-G group, with increased mMRC and CAT scores (both *p* < 0.001) and lower physical, social, and total LCQ scores (all *p* < 0.05). The BDI scores were higher in the COPD-G group (*p* = 0.034), whereas the anxiety and fatigue scores were comparable (all *p* > 0.05).

### 3.5. Findings in COPD-D, COPD-I, and COPD-E Etiotypes

Patients with COPD-D (*n* = 8) were significantly younger than those without (*p* = 0.006). No significant differences were observed in smoking exposure, pulmonary function, radiological findings, symptom burden or psychosocial outcomes (all *p* > 0.05). Overlap with COPD-C, COPD-P, COPD-I, and COPD-A was common.

Infection-related COPD (COPD-I; *n* = 42) was strongly associated with bronchiectasis, which was more prevalent than in the remaining cohort (32.5% vs. 8.1%; *p* < 0.001). Patients with COPD-I had lower smoking exposure (*p* = 0.023) and lower LCQ social scores (*p* = 0.029). No significant differences were observed in lung function, symptom burden, oxygenation, eosinophil count, or psychosocial assessment (all *p* > 0.05).

To achieve a comprehensive characterization of the predominant etiotype, patients within the COPD-E subgroup (*n* = 311) were compared with those in the non-COPD-E subgroup (*n* = 4). Patients with COPD-E exhibited significantly greater smoking exposure, with a median of 40 (30–60) pack-years compared with 0 (0–0) pack-years (*p* = 0.001). Additionally, these patients were taller (*p* = 0.017) and had higher forced vital capacity (FVC) values [2.68 (2.09–3.43) L vs. 1.67 (1.23–2.58) L; *p* = 0.042]. No statistically significant differences were identified in other clinical or functional parameters, including spirometric indices, oxygenation status, eosinophil counts, symptom burden, or psychosocial measures (all *p* > 0.05).

Although these differences were not clinically significant, this comparison provides relevant contextual information, as COPD-E represents the most prevalent etiological category in both the present cohort and the existing literature. The findings suggest that the environmental etiotype primarily reflects cumulative exposure patterns rather than a distinct physiological or psychosocial profile, underscoring the heterogeneous and exposure-driven nature of COPD in real-world clinical populations.

## 4. Discussion

In the present real-world population of patients with stable COPD, the use of the GOLD 2023 etiological taxonomy identified subclinical/integrative, functional, radiological, and psychosocial profiles in each etiotype. The most frequent etiotype observed was COPD-E, with smoking and pollution-mediated mechanisms being of paramount importance in routine clinical practice scenarios, in agreement with the key message addressed by the GOLD 2023 report [1]. In contrast, the COPD-G and COPD-I groups presented the greatest separation for airway obstruction, restriction, symptom intensity, hypoxemia, and radiological associations. The shared etiological factors in this cohort illustrate that the causes of COPD are multifactorial and provide additional support for the argument that etiotype is not completely parallel but does overlap mechanistically, if there is a pathophysiological language describing COPD heterogeneity in the current understanding [2,5].

COPD-E, that is, smoking and pollution as etiologies, was the most common cause in our study. This distribution closely mirrors the global epidemiological profile, in which tobacco smoking, biomass, and outdoor air pollution are responsible for most COPD cases [22]. The high co-occurrence of COPD-E with other etiotypes, particularly COPD-A, COPD-I, and COPD-G, suggests that the forces leading to airway injury are combined and/or interlocked. Instead of mutually exclusive pathways, these etiological profiles could reflect independent biological and environmental influences that come together. However, there seems to be a convergence to the current paradigm, in which COPD results from combined exposure and host susceptibility [7,23].

Patients with COPD-A had different demographic characteristics, such as younger age, female sex predominance, higher body mass index, and lower cumulative smoking exposure. However, pulmonary function values and most symptom-based and psychosocial scores were not significantly different from those of non-COPD-A patients, except for a higher anxiety score. These observations are consistent with those of previous studies, indicating that asthma-related mechanisms may determine the asthma–COPD overlap phenotype [24]. These data support the notion that mental health should be included as part of the standard care for patients with COPD-A and are consistent with a treatable traits framework, in which psychological distress is identified as both modifiable and clinically actionable [10,19,24].

In contrast, COPD-G has a more severe clinical expression and occurs closer to the age of onset. In those who presented with this phenotype, airflow obstruction was more severe, and hypoxemia was more prominent than in the other groups. Patients were leaner and had a higher symptom load, despite being younger. More importantly, bronchiectasis was more prevalent. This constellation of symptoms is in close agreement with the myriad of classic clinical features associated with alpha-1 antitrypsin deficiency (AATD) and other related genetic diseases, including early emphysema, profound physiological impairment, and frequent airway infections [4,22,25]. These observations are consistent with other guidelines advocating for a more routine evaluation of genetic causes, including AATD, predominantly in younger patients with severe proportional airflow obstruction [5,26].

COPD-I was significantly associated with bronchiectasis and was much more frequent than other causes. These results also support previous evidence that early life respiratory infection, post-tuberculosis lung damage, and chronic microbial colonization play a role in airway remodelling and prolonged functional loss [27,28]. Recognition of etiopathogenic lesions may facilitate targeted bidirectional strategies (airway clearance techniques, microbiological surveillance, and bronchiectasis-specific therapy) [25,29].

Various radiological patterns between etiologies also support the construct validity of the GOLD 2023 classification. COPD-A had the lowest prevalence of emphysema, whereas COPD-E had the highest. Bronchiectasis occurred more frequently in patients with COPD-G and COPD-I. The COPD-A group was also associated with non-pathological thoracic CT findings, indicating weaker structural involvement of the lungs. These trends are mechanistically plausible and indicate the potential of thoracic imaging to contribute to the etiological classification and precision imaging of COPD [30].

The heterogeneity among the etiologies in this study corresponds well with the current airway, systemic, and behavioral treatable trait domains outlined by modern COPD etiotypes [5]. In this regard, our COPD-G was mainly a systemic-based profile related to severe physiological impairment, nutritional vulnerability, and a higher risk of infection, whereas COPD-A was more associated with behavioral and psychological traits [10]. In contrast, COPD-I represents airway-centered features defined by structural abnormalities, including bronchiectasis and infection-related malfunctions. From this perspective, etiotypes could be an overarching construct for the “trait-based” clinical work framing.

Clinically, these findings indicate that etiological assessment may be informative in addition to traditional spirometric classification. Radiological investigations, including the detection of bronchiectasis, could be useful in patients with COPD-I and COPD-G (especially). A systematic psychological examination may be more informative for patients with COPD-A. In addition, severe airflow limitation in young patients should raise awareness of any genetically predisposing factors and check for underlying causes, such as AATD [7]. Taken together, these observations would support a multidimensional approach to evaluation in which etiological stratification guides the prioritization of focused evaluations rather than displacing traditional COPD evaluation paradigms.

This study has several limitations. First, the cross-sectional and single-center design constrains the ability to establish causal relationships and may impact the generalizability of the findings. Second, the sample sizes of certain etiotype subgroups, particularly COPD-G and COPD-D, were relatively small, potentially diminishing the statistical power to detect significant differences and limiting the feasibility of conducting multivariate analyses that adjust for potential confounders such as age, sex, and smoking exposure. Third, certain variables, including smoking exposure and psychosocial assessments, were based on patient self-reports and may be susceptible to recall bias. Finally, the study did not incorporate longitudinal follow-up to evaluate clinical outcomes or disease progression across etiologies. Future multicenter prospective studies with larger and more balanced samples are required to validate and extend these findings.

## 5. Conclusions

In summary, the GOLD 2023 etiological taxonomy is linked to distinct clinical, functional, radiological, and psychosocial phenotypes in patients with stable COPD. This study offers a comprehensive real-world evaluation of the GOLD 2023 cause-based classification, illustrating that consistent etiotype-specific patterns, along with frequent etiological overlap, reinforce the mechanistic foundation of the taxonomy and its utility as a multidimensional framework for COPD characterization. From a practical clinical standpoint, etiological stratification may aid clinicians in prioritizing diagnostic investigations and adopting more individualized management strategies beyond conventional spirometric classification. These findings highlight the clinical significance of etiological stratification and support its integration into personalized, trait-based approaches for COPD management.

## Figures and Tables

**Table 1:** Baseline Characteristics of the Study Population (*n* = 315).

Variable	Descriptive Statistics
Age (years), mean ± SD	64.2 ± 10.8
Female sex, *n* (%)	39 (12.4)
Current smokers, *n* (%)	157 (49.8)
Former smokers, *n* (%)	135 (42.9)
Never smokers, *n* (%)	16 (5.1)
Smoking exposure (pack-years), median (P25-P75)	40 (30–60)
Family history of COPD, *n* (%)	71 (22.5)
Oxygen saturation ≥ 90%, *n* (%)	294 (93.3)
Eosinophil count ≥ 300/μL, *n* (%)	70 (22.2)
Thoracic CT performed, *n* (%)	263 (83.5)
Any pathological finding on CT, *n* (%)	252 (95.8)
GOLD severity classification, *n* (%)	
• Mild (GOLD I)	47 (14.9)
• Moderate (GOLD II)	150 (47.6)
• Severe (GOLD III)	88 (27.9)
• Very severe (GOLD IV)	30 (9.5)

Abbreviations: COPD = Chronic obstructive pulmonary disease; GOLD = Global Initiative for Chronic Obstructive Lung Disease; SD = Standard deviation; CT = Computed tomography.

**Table 2:** Distribution and Overlap of GOLD 2023 Etiotypes and Subgroup Intersections.

Etiotype	N	Subgroup Intersections	*n*
COPD-G	8	G ∩ A	1
		G ∩ I	1
		G ∩ C ∩ P	5
		G ∩ D ∩ P ∩ I	1
COPD-D	8	D ∩ C ∩ A	1
		D ∩ C ∩ I	1
		D ∩ C ∩ P	2
		D ∩ C ∩ P ∩ A	1
		D ∩ C ∩ P ∩ I ∩ A	1
		Additional D combinations	2
COPD-A	31	A (only)	1
		A ∩ C	3
		A ∩ P	1
		A ∩ I	1
		A ∩ P ∩ C	19
		A ∩ C ∩ D	1
		A ∩ G	1
		A ∩ P ∩ I ∩ C	1
		A ∩ C ∩ P ∩ D	1
		A ∩ C ∩ P ∩ I ∩ D	1
COPD-I	42	I ∩ C	3
		I ∩ P	1
		I ∩ P ∩ C	31
		I ∩ A	1
		I ∩ G	1
		I ∩ C ∩ D	1
		I ∩ P ∩ C ∩ A	1
		I ∩ D ∩ P ∩ G	1
COPD-E (COPD-C + COPD-P)	311	C (only)	27
		P (only)	2
		C ∩ P	209
		C ∩ P ∩ I	31
		C ∩ P ∩ A	19
		C ∩ P ∩ D	2
		C ∩ P ∩ G	5
		C ∩ A	3
		C ∩ I	3
		C ∩ P ∩ I ∩ A	1
		C ∩ P ∩ D ∩ I ∩ A	1
COPD-U	0	-	0

Subgroup intersections represent individuals who fulfill the criteria for more than one etiotype. Because etiotypes are etiological—not mutually exclusive—categories, the total may exceed 315. Abbreviations: COPD: Chronic obstructive pulmonary disease, COPD-A: Asthma-associated COPD, COPD-C: Smoking-related COPD, COPD-D: Development-related COPD, COPD-E: Environmental exposure–related COPD, COPD-G: Genetically determined COPD, COPD-I: Infection-related COPD, COPD-P: Biomass/pollution-related COPD, COPD-U: Unclassified COPD.

**Table 3:** Demographic, functional, laboratory, and symptom-related characteristics of patients with COPD-A and non-COPD-A.

Variables	Non-COPD-A (*n* = 284)	COPD-A (*n* = 31)	Test Statistic (Z/t/χ^2^)	*p*
Demographic				
Age (years)	66 (59–72)	54 (47–60)	−5.375	**<0.001**
Female Male	26 (9.2)	13 (41.9)	0.000	**<0.001**
	258 (90.8)	18 (58.1)		
BMI (kg/m^2^)	25.39 (22.67–29.06)	27.34 (24.76–33.27)	–2.380	**0.017**
Smoking (pack/years)	40 (30–60)	30 (0–50)	–3.330	**<0.001**
Functional				
FVC (L)	2.67 (2.04–3.36)	2.70 (2.15–3.73)	–0.549	0.583
FVC (% predicted)	77 (64–91.75)	79 (64–93)	–0.518	0.604
FEV_1_ (L)	1.52 (1.10–2.00)	1.64 (1.24–2.11)	–1.022	0.307
FEV_1_ (% predicted)	57.17 ± 20.12	58.87 ± 19.12	–0.448	0.654
FEV_1_/FVC (%)	59 (51–65)	62 (55–65)	–0.924	0.355
Laboratory				
Hemoglobin (g/dL)	14.30 (13.40–15.30)	14.70 (13.80–15.40)	–1.278	0.201
Eosinophils (%)	2.30 (1.30–3.60)	2.40 (1.00–2.70)	–0.758	0.448
Eosinophils (μL)	190 (102.5–270)	210 (100–260)	–0.390	0.697
SpO_2_ (room air, %)	96 (94–97)	97 (94–98)	–1.056	0.291
SpO_2_ (with O_2_ support, %)	93.65 ± 2.29	95 ± 2	–0.967	0.356
Symptomatical				
mMRC score	1 (1–1.75)	1 (1–1)	–0.388	0.698
CAT score	6 (3–12)	8 (4–16)	–1.117	0.264
LCQ Physical	6.37 (5.62–6.75)	6.25 (5.00–6.62)	–1.304	0.192
LCQ Psychological	6.71 (6–7)	6.71 (6.14–7)	–0.064	0.949
LCQ Social	7 (6.75–7)	7 (6.50–7)	–0.569	0.569
LCQ Total	20.13 (18.18–20.60)	19.71 (17.12–20.50)	–0.774	0.439
BDI	5 (2–10)	7 (3–13)	–0.802	0.423
BAI	4 (1–7)	6 (3–9)	–2.583	**0.010**
CAFS	16.66 (4.16–31.25)	16.66 (4.16–31.25)	–0.750	0.453

Z values correspond to the Mann–Whitney U test, t values to the independent samples *t*-test, and χ^2^ values to the chi-square tests. Data are presented as median (P25-P75), mean ± SD, or *n* (%). Bold *p*-values indicate statistical significance. Abbreviations: BMI: Body Mass Index, FEV_1_: Forced expiratory volume in 1 s, FVC: Forced vital capacity, SpO_2_: Peripheral oxygen saturation, mMRC: Modified Medical Research Council Dyspnea Scale, CAT: COPD assessment test, LCQ: Leicester cough questionnaire, BDI: Beck depression inventory, BAI: Beck anxiety inventory, CAFS: COPD–Asthma Fatigue Scale.

**Table 4:** Demographic, functional, laboratory, and symptom-related characteristics of patients with COPD-G and non-COPD-G.

Variables	Non-COPD-G (*n* = 307)	COPD-G (*n* = 8)	Test Statistic (Z/t/χ^2^)	*p*
Demographic				
Age (years)	66 (58–72)	48.50 (31.75–53.25)	−4.108	**<0.001**
Female Male	37 (12.1)	2 (25)	0.259	0.259
	270 (87.9)	6 (75)		
BMI (kg/m^2^)	25.66 (22.86–29.38)	22.03 (20.44–25.64)	–2.064	**0.039**
Smoking (pack/years)	40 (30–60)	12.50 (0–37.50)	–3.065	**0.002**
Functional				
FVC (L)	2.69 (2.10–3.43)	1.78 (1.68–1.80)	–2.554	**0.011**
FVC (% predicted)	77 (64–92)	55 (39.25–58.75)	–2.954	**0.003**
FEV_1_ (L)	1.58 (1.14–2.00)	0.72 (0.58–1.03)	–3.012	**0.003**
FEV_1_ (% predicted)	57 (44–71)	25.50 (19–28.75)	–3.465	**<0.001**
FEV_1_/FVC (%)	60 (52–65)	40 (32.50–62.75)	–2.196	**0.028**
Laboratory				
Hemoglobin (g/dL)	14.30 (13.50–15.30)	13.40 (10.65–15.78)	–1.253	0.210
Eosinophils (%)	2.30 (1.30–3.50)	2.20 (0.40–3.00)	–0.755	0.450
Eosinophils (μL)	190 (100–270)	185 (45–300)	–0.338	0.735
SpO_2_ (room air, %)	96 (94–98)	85.50 (84.25–93.75)	–3.206	**0.001**
SpO_2_ (with O_2_ support, %)	93.65 ± 2.29	92.80 ± 1.79	0.757	0.458
Symptomatical				
mMRC score	1 (1–1)	3.5 (2.25–4)	–3.620	**<0.001**
CAT score	6 (3–12)	21 (13.50–27.50)	–3.782	**<0.001**
LCQ Physical	6.37 (5.62–6.75)	5.44 (4.56–6.38)	–2.218	**0.027**
LCQ Psychological	6.71 (6–7)	5.85 (3.28–6.82)	–1.842	0.066
LCQ Social	7 (6.75–7)	6 (4.75–7)	–2.630	**0.009**
LCQ Total	20.14 (18.21–20.60)	17.33 (12.51–20.07)	–2.192	**0.028**
BDI	5 (2–10)	11 (7–22.50)	–2.125	**0.034**
BAI	4 (1–7)	5.5 (3.25–13.75)	–1.680	0.093
CAFS	16.66 (4.16–31.25)	26.04 (11.97–67.18)	–1.467	0.142

Z values correspond to the Mann–Whitney U test, t values to the independent samples *t*-test, and χ^2^ values to the chi-square tests. Data are presented as median (P25-P75), mean ± SD, or *n* (%). Bold *p*-values indicate statistical significance. Abbreviations: BMI: Body Mass Index, FEV_1_: Forced expiratory volume in 1 s, FVC: Forced vital capacity, SpO_2_: Peripheral oxygen saturation, mMRC: Modified Medical Research Council dyspnea Scale, CAT: COPD assessment test, LCQ: Leicester cough questionnaire, BDI: Beck depression inventory, BAI: Beck anxiety inventory, CAFS: COPD–Asthma Fatigue Scale.

## Data Availability

Data supporting the findings of this study are available from the corresponding author upon reasonable request. The data were not publicly available because of ethical and privacy restrictions related to the patient’s confidentiality.

## Abbreviations

The following abbreviations are used in this manuscript.

COPD	Chronic obstructive pulmonary disease
COPD-A	Asthma-associated COPD
COPD-C	Smoking-related COPD
COPD-D	Development-related COPD
COPD-E	Environmental exposure–related COPD
COPD-G	Genetically determined COPD
COPD-I	Infection-related COPD
COPD-P	Biomass/pollution-related COPD
COPD-U	Unclassified COPD
GOLD	Global Initiative for Chronic Obstructive Lung Disease
mMRC	Modified Medical Research Council dyspnea Scale
CAT	COPD assessment test
LCQ	Leicester cough questionnaire
BDI	Beck depression inventory
BAI	Beck anxiety inventory
CAFS	COPD–Asthma Fatigue Scale
FEV_1_	Forced expiratory volume in 1 s
FVC	Forced vital capacity
SpO_2_	Peripheral oxygen saturation
CT	Computed tomography
AAT	Alpha-1 antitrypsin
AATD	Alpha-1 antitrypsin deficiency
BMI	Body mass index
